# Identifying Alzheimer’s disease-related pathways based on whole-genome sequencing data

**DOI:** 10.1016/j.csbj.2025.09.013

**Published:** 2025-09-12

**Authors:** Yongheng Wang, Taihang Liu, Yijie He, Yaqin Tang, Pengcheng Tan, Lin Huang, Dongyu Huang, Tong Wen, Lizhen Shao, Jia Wang, Yingxiong Wang, Zhijie Han

**Affiliations:** a Department of Bioinformatics, School of Basic Medicine, Chongqing Medical University, Chongqing, China; b Department of Laboratory Medicine, Chongqing Jiulongpo People's Hospital, Chongqing, China; c Key Laboratory of Major Brain Disease and Aging Research (Ministry of Education), Chongqing Medical University, Chongqing, China; d Institute for Brain Science and Disease, Chongqing Medical University, Chongqing, China

**Keywords:** Alzheimer’s disease, Pathway, Polygenic risk score, Single sample gene set variation analysis, Structural equation modeling

## Abstract

Alzheimer’s disease (AD) is a highly inheritable neurodegenerative disorder for which pathway-specific genetic profiling provides insights into its key biological mechanisms and potential treatment targets. Traditional disease-pathway analyses for AD have certain limitations, such as environmental interference and arbitrary sample division. We present a comprehensive framework that starts with genome data, avoiding these drawbacks and offering intrinsic pathway-specific genetic profiling for AD. Whole genome sequencing data from 173 individuals were used to quantify transcriptomes in 14 brain regions, estimate individual-level pathway variant scores, and analyze AD risk for each patient. These results were combined to identify AD-related pathways and quantify their interactions. The predicted expression levels were consistent with previous findings, and the estimated AD risk showed a significant correlation with Braak/Thal scores. A total of 3798 pathways were identified as potentially associated with AD, with about 19.7 % previously reported. The pathways identified as AD risk related primarily address six core biological themes, including: Immunity and inflammation, Metabolism, Protein homeostasis, DNA/RNA and Epigenetics, Synapse and structure, Cell cycle. Specifically, key pathways, such as NF-κB signaling and GSK3β activation, were linked to AD pathogenesis. The interactions among pathways highlighted shared gene functions in AD. In summary, we provided an effective framework for disease-pathway analysis, revealing the interdependence or compensatory effects of pathways in AD.

## Highlights

• This study presented a novel comprehensive framework starting from genome data to identify the Alzheimer’s disease (AD)-related pathways.
• The framework can overcome the limitations of traditional disease-pathway analyses for AD, such as environmental interference and arbitrary sample division.
• The largest number of novel pathways related to AD was identified, and numerous previously reported findings were confirmed.
• The interactions among pathways emphasized shared gene functions in AD.

## Graphical Abstract

**Figure:**
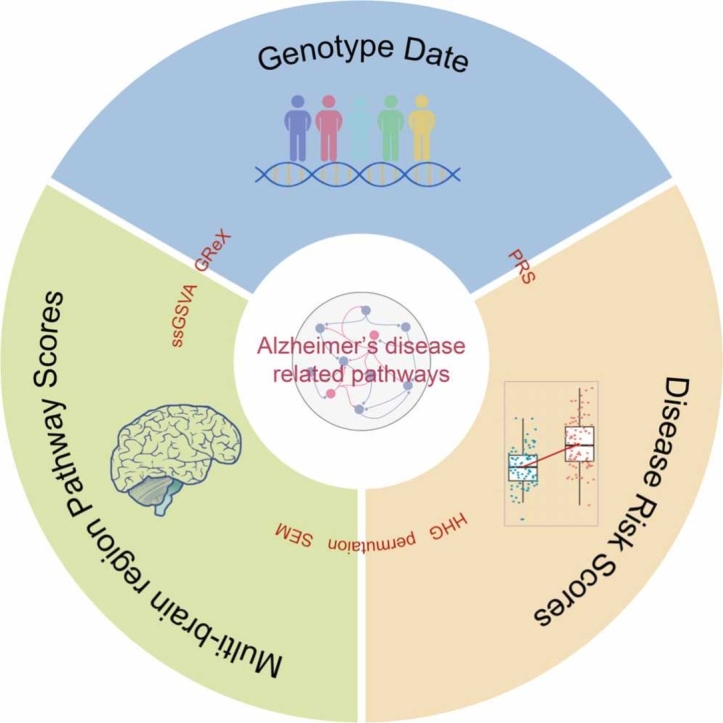

## 1 Introduction

Alzheimer’s disease (AD) is a progressive neurodegenerative disorder with a high heritability estimated between 60 % and 80 % [1]. Despite the identification of numerous genetic variants associated with AD risk and pathology [2], [3], [4], [5], its precise pathogenesis is not fully understood, and effective clinical treatments are still lacking. Pathway-specific genetic analysis offers a powerful approach to understand the key biological mechanisms involved in diseases and enables the identification of causal genes and potential therapeutic targets [6]. Therefore, studying the genetic characteristics of AD-related pathways is essential. Typically, the protocol for inferring disease-related pathways relies on detecting differential characteristics between disease and control groups. For example, a case-control-based genotype-phenotype association analysis highlighted the involvement of amyloid/tau pathways in AD [7]. Furthermore, analyses of transcriptomic [8], [9] and proteomic [10], [11] differences between AD patients and controls have uncovered several new AD-related pathways.

However, the case-control design has several key limitations: (1) These analyses require artificially dividing samples into disease and control groups. For a progressive disease like AD, establishing a progressive state of risk and severity, it is challenging to establish a clear standard for distinguishing whether a person is diseased or not. For example, early AD diagnosis relies on assessing AD neuropathological changes, including neurofibrillary tangle distribution (Braak NFT stage), amyloid-beta plaques (Thal stage), and the neuronal plaques density in the neocortex (CERAD score). In the preclinical (asymptomatic) phase of the AD continuum, misdiagnosis and missed diagnosis may occur [12]. Additionally, the observed phenotype of AD patients (e.g., behavioral rating scale) and their pathological diagnosis (e.g., senile plaque and neurofibrillary tangle pathology) can sometimes be contradictory [13]. (2) Analysis based on quantifying expression levels, which can be influenced by several factors such as cell type heterogeneity effects and environmental factors, leading to potentially false associations with disease pathogenesis. This includes incorrect findings of blood and cerebrospinal fluid-based biomarkers used for early AD diagnosis [14]. (3) Even considering influence factors, determining disease-related genes and pathways is often based on a one-size-fits-all threshold, which may result in losing a significant amount of potentially relevant gene information.

Genomic information provides valuable insights into the original genetic characteristics and the molecular basis of pathogenesis. Disease-related single nucleotide polymorphisms (SNPs), genes, and individual disease risks can be identified from genomic data using methods like genome-wide association studies (GWAS), fine-mapping, linkage disequilibrium score regression (LDSC), and polygenic risk score (PRS) [15], [16]. Compared to single SNP analyses, gene and gene set analyses are considered more powerful [17]. Gene set analysis, in particular, offers valuable insights into the role of specific biological pathways or cellular functions in the genetic basis of phenotypes [18]. While pathway-based PRS tools like PRSet [19], MAGMA [18], and stratified LDSC [17] exist, they typically only assign SNPs to genes, then genes to gene sets, and construct test statistics. These tools link variants to pathways based on their location, without considering the correlation between SNPs and gene expression levels, which are captured by expression quantitative trait loci (eQTL).

To address these limitations, this study developed a novel framework that investigate the genetic characteristics of AD pathways directly from the genome. Our approach successfully identified pathways related to AD across different brain regions based on their genetic characteristics. The advantages of our analysis are noteworthy: it requires only genotyping data (obtaining brain tissue is challenging, but blood samples are easily accessible), does not require discrete case-control labels and can analyze continuous phenotypes. By starting our analysis directly from the genome, our framework provides results that reflect primary genetic characteristics, minimizing potential confounding from downstream biological or environmental factors. We believe this framework holds promise for providing quantitative insights into the combined effects between pathways in complex diseases.

## 2 Results

### 2.1 Pipeline overview

**Fig. 1:**
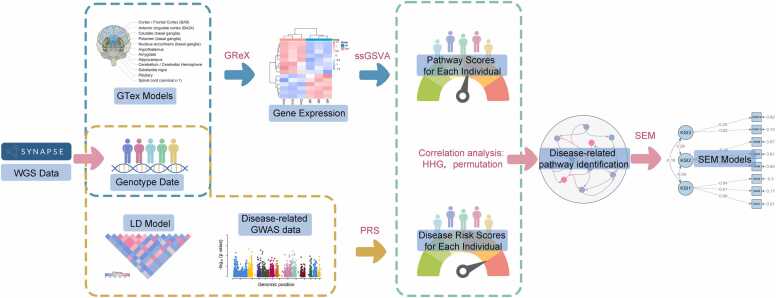
Pipeline schematic of investigating the original genetic characteristics of AD pathways. In this pipeline, only genotype data is required, which can be obtained through various methods such as WGS, whole exome sequencing (WES), or SNP array analysis. Pathway scores and disease risk scores for each individual were calculated based on their genotype data. Subsequently, analyses were conducted to explore AD-related pathways and the interaction models between these pathways. This genotype data-driven approach eliminates potential confounding factors that influence gene expression levels, allowing for a focused investigation into genetic regulation. Furthermore, this approach can also be adapted for analyzing genetic regulatory pathways in other diseases.

To investigate the genetic characteristics of AD, we used whole genome sequencing (WGS) data from 173 individuals (including 92 AD cases and 81 controls according to the original annotations) obtained from the MayoRNAseq (https://doi.org/10.7303/syn10901601). Since most associations between genes/pathways and diseases are tissue-specific [20], we first used a method called genetically regulated component of expression (GReX) to produce genome-wide expression data from 14 brain regions of GTEx (v8) [21]. This genotype data-based approach eliminates potential confounding factors that affect gene expression levels, such as environmental influences, allowing us to focus on the genetic regulation [22]. We then used the quality-controlled genotype data to estimate individual AD risk scores by PRS, instead of categorizing individuals based on original annotations. Subsequently, we performed a single sample gene set variation analysis (ssGSVA) to analyze individual-level pathway variant scores based on quantified gene expression [23]. We combined these scores with PRS to identify AD-related pathways through a distance rank correlation analysis [24]. Finally, we conducted a structural equation modeling (SEM) analysis to quantify interactions among the AD-related pathways, identifying possible genetic etiology and pathway mechanisms of AD (Fig. 1).

### 2.2 Transcriptome prediction and gene sets variation analysis based on genotype data

The total measured expression level of a gene (e.g. obtained through RNA-seq) can be conceptually divided into the following parts:

Total Measured Expression = Genetically Regulated Expression + Non-heritable Genetic Effects + Environmental Effects + Cell type Heterogeneity Effects + Stochastic Noise + Technical Artifacts

**Fig. 2:**
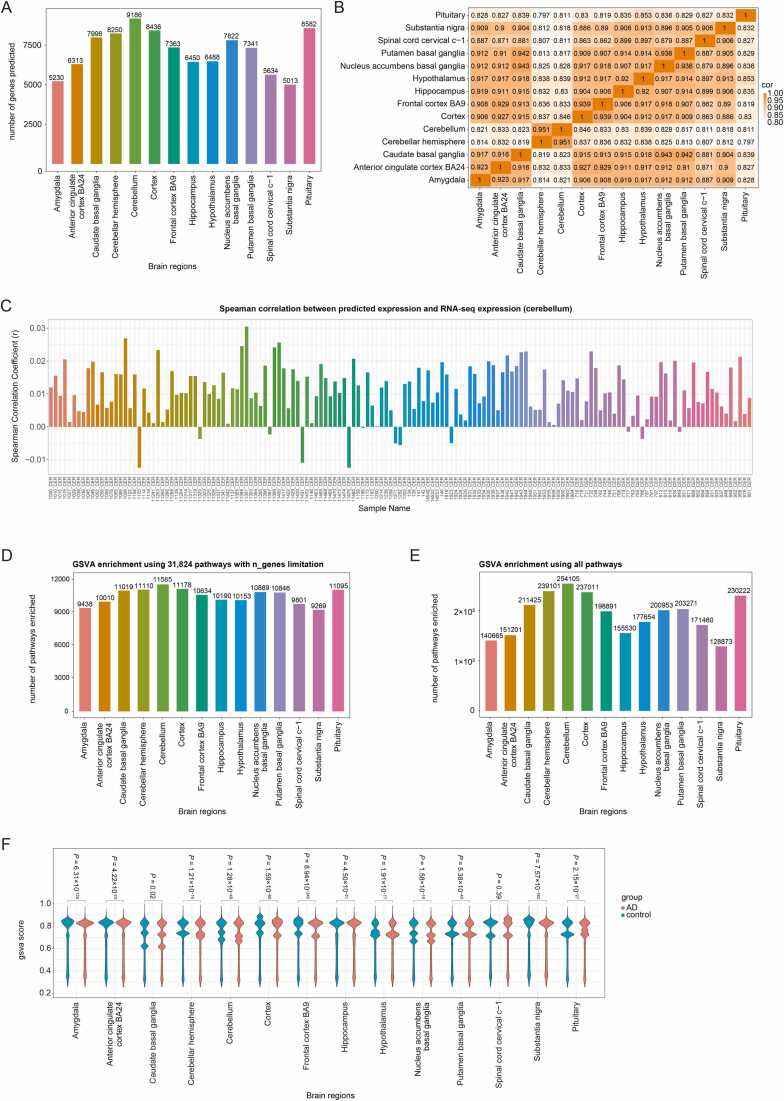
Performance of GReX and ssGSVA. (**A**) The number of genes predicted by GReX method for the 14 brain regions. (**B**) The pairwise correlations computed using the Pearson method among 14 brain regions based on the predicted expression levels. (**C**) The spearman correlation between predicted expression profile and RNA-seq gene expression data of cerebellum region. (**D**) The number of pathways with ssGSVA score enriched by ssGSVA method using the 31,824 pathways with number of genes limitation in 10–300 for the 14 brain regions. (**E**) The number of pathways with ssGSVA score enriched by ssGSVA method using all pathways for the 14 brain regions. (**F**) The distribution of ssGSVA scores in AD and control samples.

This research focus on the original genetic characteristics of AD, the Genetically Regulated Expression part. To measure this part, we predicted the gene expression from the genomic data by GReX method [22]. From the 349 samples in the MayoRNAseq WGS study, we selected 92 AD samples and 81 control samples, all of White individuals. The results of principal component analysis (PCA) and Tracy-Widom statistical test (*P* > 0.05) showed that there was no significant population structure in the WGS data. Based on the genomic features, we predicted transcriptome expression in 14 brain regions from GTEx (v8), including: amygdala, anterior cingulate cortex BA24, caudate basal ganglia, cerebellar hemisphere, cerebellum, cortex, frontal cortex BA9, hippocampus, hypothalamus, nucleus accumbens basal ganglia, putamen basal ganglia, spinal cord cervical c-1, substantia nigra, and pituitary. As shown in Fig. 2**A**, the number of predicted genes varied, ranging from 5013 in substantia nigra to 9186 in cerebellum. The predicted gene expression levels ranged from −3–3. Furthermore, pairwise correlation analysis of brain regions revealed that gene expression correlations between the cerebellum, cerebellar hemisphere, and pituitary, and other brain regions were all below 0.85 (Fig. 2**B**). In contrast, correlations between other brain regions were relatively high. The correlation between similar brain regions exceeded 0.93, including the caudate basal ganglia, nucleus accumbens basal ganglia, and putamen basal ganglia; the cortex and frontal cortex BA9; and the cerebellar hemisphere and cerebellum. These results align with previous findings that gene expression regulation shows specificity in the cerebellum and similarity across various regions of the cerebral cortex [25].

In addition, we evaluated the correlation between predicted expression profile and RNA-seq gene expression data of cerebellum region, revealing a weak but expected association (range: −0.012–0.031; Fig. 2C). The correlations are associated with the expected performance of the model. Then we summarized the prediction performance using GTEx v8. As shown in Supplementary Table S1, for ∼50 % of the genes, either in the cerebellum or across all 14 brain regions, the model explained less than 10 % of the expression variance (R2). For ∼75 % of the genes, the variance explained was less than 20 %. The median R2 was 0.095 for cerebellum and 0.083 for all regions, while mean values were 0.148 and 0.132, respectively. The mean values were higher than the medians, indicating a right-skewed distribution with most genes showing low explanatory power.

Further, to identify AD-related pathways, we performed non-parametric, unsupervised ssGSVA [23] analysis using gene sets from multiple resources, including GSEA [26], DAVID [27], Reactome [28], NetPath [29], PANTHER [30], WikiPathways [31], and PathBank database [32], totaling 1649,335 pathways. The type, number, version and detail description information of the pathways is shown in Supplementary Table S2. We firstly focused on 31,824 commonly used or well-annotated pathways, including type: GOBP, GOCC, COMF, KEGG, Reactome, WikiPathways, and four types from MSigDB (CP, CP:BIOCARTA, CP:PID, IMMUNESIGDB) that are worth paying attention to in AD research. The ssGSVA scores were calculated for each individual across 14 brain regions, and the parameter for the number of genes (n_genes) in the pathway was set to 10–300 to make the enrichment results more reliable. As shown in Fig. 2**D**, the number of detectable pathways ranged from 9269 (substantia nigra) to 11,585 (cerebellum). For comprehensive screening, we enriched using all 1649,335 pathways with a loose parameter n_genes > 1. As shown in Fig. 2**E**, the number of pathways range expanded to 128,873 (substantia nigra) and 254,105 (cerebellum). These trends are consistent with the trend observed in the number of predicted genes. Additionally, we calculated the average ssGSVA score when using all pathways among the originally annotated AD cases and controls, respectively, and compared their distribution in the 14 brain regions using a two-tailed Wilcoxon test (the threshold of *P* < 0.05). We observed significant differences in most brain regions, except the spinal cord cervical c-1 (*P* = 0.39) (Fig. 2**F**).

### 2.3 Identification of AD risk associated pathways

**Fig. 3:**
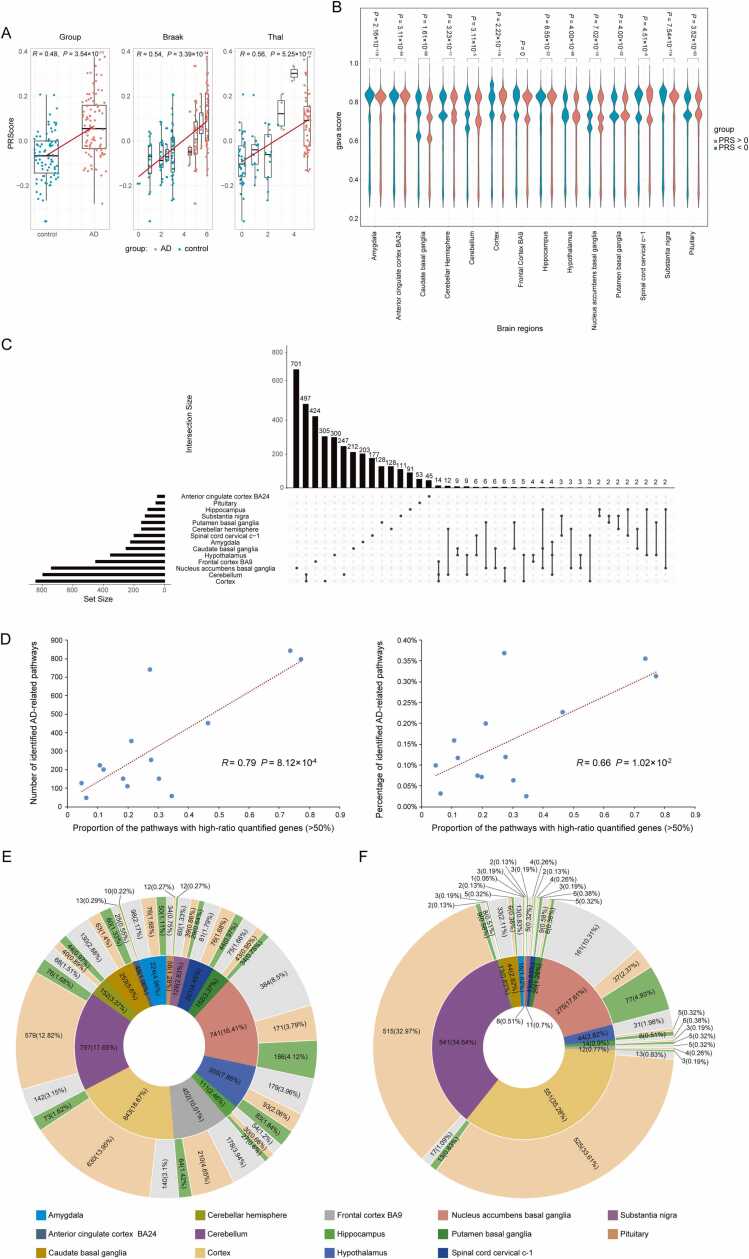
Performance of PRS and identification of AD-related pathways. (**A**) Spearman correlation analysis depicting the association between AD risk score (measured by PRS analysis) and AD/control group, Braak stage, and Thal stage. (**B**) The distribution of ssGSVA scores in high (more than zero) and low (less than zero) AD risk samples. (**C**) Visualization of intersecting sets of AD-related pathways identified by HHG analysis and permutation procedure. (**D**) The relationship between the proportion of pathways with high-ratio quantified genes and the efficiency of AD-related pathway identification. (**E**) The proportion of direct (green), indirect (burlywood) and unrelated (grey) pathways within each brain region among all AD risk-related pathways and (**F**) among all pathways in SEM results.

To estimate the genetic liability to AD risk in individuals, we performed PRS analysis. Following PRS guidelines, we retained 173 samples (92 AD cases and 81 controls according to the original annotations) and 10,070,779 SNPs after applying filters and quality control (QC). By combining with the QCed AD GWAS summary data based on Jansen et al. [33], we calculated an AD risk score for these 173 individuals, known as the PRS. We assessed the similarity between the AD risk scores and the original annotations by performing linear Pearson's analysis. Despite some differences, the AD risk score showed a significant correlation with individual group (*R* = 0.48, *P* = 3.54 ×10^−11^), Thal stage (*R* = 0.54, *P* = 3.39 ×10^−14^), and Braak NFT stage (*R* = 0.56, *P* = 5.25 ×10^−12^) (Fig. 3**A**). We also compared the distribution of the average ssGSVA scores between individuals with high (more than zero) and low (less than zero) AD risk scores in the 14 brain regions using the two-tailed Wilcoxon test. Significant differences were found in all 14 brain regions (*P* < 0.05), and these differences were more pronounced overall compared to those observed in the previous step (grouping by original annotations) (Fig. 3**B**).

Further, we identified the AD risk associated pathways. Since most ssGSVA scores do not follow a normal distribution (Shapiro-Wilk test, *P* < 0.05), and the correlation pattern between ssGSVA scores and AD risk scores was unknown, we employed the Heller-Heller-Gorfine (HHG) method to assess the correlation [34], [35]. Before conducting the correlation analysis, we removed outliers from the scores. Finally, the AD-related pathways were identified through a permutation procedure. It is worth noting that the significant correlation pathways identified by HHG (*P* < 0.001) matched those identified by the permutation procedure. As results, 252 pathways were identified as significantly associated with the AD risk score when using 31,824 pathways with n_genes limitation. Pathways identified in each brain region are listed in Supplementary Table S3. As summarized in Table 1, the pathways primarily involve six core pathological characteristics, including Immunity and inflammation, Metabolism, Protein homeostasis, DNA/RNA and Epigenetics, Synapse and structure, and Cell cycle, among which immunity and inflammation represent the foremost pathological feature. Moreover, 3798 pathways were significantly associated with the AD risk using all pathways, and the 252 pathways are fully included in the expanded 3798 pathways. The pathways also include these 6 core pathological features (Table 2). The expanding data demonstrate that AD is a complex network disease affecting multiple systems throughout the brain. Since we focus on the all AD-related pathways, the expanded 3798 pathways were used for subsequent analysis. Table 3 demonstrates that the number of pathways significantly related to AD risk in each brain region, ranged from 48 (anterior cingulate cortex BA24) to 843 (cortex). Moreover, pathways were identified in at least two brain regions accounting for about 16 % of the total (Fig. 3**C**). In addition, we define a pathway in which more than 50 % of genes are quantified by GReX and used for ssGSVA scoring as a pathway with high-ratio quantified genes. We observed that the more pathway-related genes quantified in a tissue, the more AD-related pathways were identified within it. Fig. 3**D** shows a significant positive correlation between the proportion of pathways with high-ratio quantified genes and the percentage of identified AD-related pathways (*R* = 0.79, *P* = 8.12 ×10^−4^). This suggests that more comprehensive eQTL data can enhance the efficiency of our framework.

**Table 1:** Overview of the pathways predicted in 14 brain regions when using 31,824 pathways with the number of genes limitation in: 10–300.

Brain region	Immunity and inflammation	Metabolism	Protein homeostasis	DNA/RNA and Epigenetics	Synapse and structure	Cell cycle	Other	Total
Amygdala	15	0	1	3	0	0	1	20
Anterior cingulate cortex BA24	1	0	0	0	0	0	0	1
Caudate basal ganglia	12	0	0	1	1	2	8	24
Cerebellar hemisphere	7	0	0	1	0	0	3	11
Cerebellum	13	1	2	0	1	0	5	22
Cortex	15	2	1	1	1	0	8	28
Frontal cortex BA9	15	0	3	6	4	1	6	35
Hippocampus	4	0	1	3	0	0	0	8
Hypothalamus	17	2	3	1	1	1	7	32
Nucleus accumbens basal ganglia	19	4	1	1	4	0	8	37
Putamen basal ganglia	7	3	0	0	0	0	0	10
Spinal cord cervical c−1	8	2	0	1	1	0	2	14
Substantia nigra	8	0	1	1	1	0	1	12
Pituitary	2	0	0	0	1	0	1	4

**Table 2:** Overview of the pathways predicted in 14 brain regions when using all pathways.

Brain region	Immunity and inflammation	Metabolism	Protein homeostasis	DNA/RNA and Epigenetics	Synapse and structure	Cell cycle
Amygdala	25	14	9	39	10	29
Anterior cingulate cortex BA24	2	4	1	7	2	16
Caudate basal ganglia	23	19	12	28	5	28
Cerebellar hemisphere	14	10	6	18	8	21
Cerebellum	18	22	15	31	11	45
Cortex	24	18	14	35	20	52
Frontal cortex BA9	22	12	10	33	15	38
Hippocampus	11	8	7	15	6	12
Hypothalamus	23	16	18	30	9	35
Nucleus accumbens basal ganglia	19	15	11	29	10	31
Putamen basal ganglia	15	11	8	21	7	25
Spinal cord cervical c−1	5	3	2	6	4	8
Substantia nigra	20	14	10	26	9	29
Pituitary	10	7	9	14	5	13

**Table 3:** Overview of the genes and pathways predicted in 14 brain regions when using all pathways with the number of genes > 1.

Brain region	Pathway				Gene		
	**ssGSVA**	**AD risk- related**	**Literature-reported AD-related**	**Percentage**^**a**^	**predicted**	**In literature-reported AD pathways**	**Percentage**^**b**^
**Amygdala**	140665	224	50	22.32 %	5230	1038	19.85 %
**Anterior cingulate cortex BA24**	151201	48	10	20.83 %	6313	160	2.53 %
**Caudate basal ganglia**	211425	253	59	23.32 %	7998	1015	12.69 %
**Cerebellar hemisphere**	239101	152	44	28.95 %	8250	1007	12.21 %
**Cerebellum**	254105	797	76	9.54 %	9186	906	9.86 %
**Cortex**	237011	843	72	8.54 %	8436	4078	48.34 %
**Frontal cortex BA9**	198891	452	64	14.16 %	7363	786	10.67 %
**Hippocampus**	155530	111	27	24.32 %	6450	431	6.68 %
**Hypothalamus**	177654	355	83	23.38 %	6488	2494	38.44 %
**Nucleus accumbens basal ganglia**	200953	741	187	25.24 %	7822	1567	20.03 %
**Putamen basal ganglia**	203271	152	34	22.37 %	7341	651	8.87 %
**Spinal cord cervical c−1**	171460	201	43	21.39 %	5634	398	7.06 %
**Substantia nigra**	128873	128	29	22.66 %	5013	254	5.07 %
**Pituitary**	230222	58	12	20.69 %	8582	109	1.27 %

Percentage^a^: number of literature-reported AD-related pathways/number of AD risk-related pathways. Percentage^b^: number of genes in literature-reported AD-related pathways/number of predicted genes.

### 2.4 Literature reported relation between AD risk-related pathways and AD

To verify the relevance of the identified pathways to AD, we investigated the literature-reported correlation between the 3798 AD risk-related pathways identified across 14 brain regions and AD. These correlations were classified as direct, indirect (associated with features linked to AD), or unrelated (Detailed definition scheme is in Methods). Table 3 provides the number of identified AD risk-related pathways, literature-reported directly AD-related pathways, and the percentage of direct correlations within each brain region. Detailed descriptions of the reported relevance between the enriched pathways and AD are listed in Supplementary Table S4, along with corresponding references. Additionally, Fig. 3**E** illustrates the proportion of direct, indirect, and unrelated pathways within each brain region among all AD risk-related pathways. The pathways directly correlated with AD widely distributed in important brain regions related to cognition, memory, and motor function, such as the cortex, hippocampus, basal ganglia, and cerebellum. The NF-κB signaling pathway, a core neuroinflammatory pathway, and its related molecules has been found to be associated with AD in multiple brain regions, including the frontal cortex BA9, putamen basal ganglia, nucleus accumbens basal ganglia, substantia nigra, and cervical spinal cord c-1. TLR4 and TRAF6, as upstream molecules of NF-κB, are both significantly enriched in the cortex. GSK3β (glycogen synthase kinase 3β), a key kinase regulating tau phosphorylation, is involved in the cortex, cerebellum, and basal ganglia. Tau protein: As one of the core pathological proteins in AD, its abnormal phosphorylation and aggregation have been studied in multiple brain regions, including the cortex, cerebellum, amygdala, and nucleus accumbens basal ganglia. β-catenin and Wnt signaling pathways: This pathway is closely related to neuronal function and is enriched in the cortex, cerebellar hemisphere, hypothalamus, and amygdala. The PI3K/AKT signaling pathway: This is a key cell survival and metabolic pathway, expressed in hypothalamus, substantia nigra, and caudate basal ganglia. MACF1 (microtubule-actin cross-linking factor 1), as a molecule that directly affects microtubule stability, its expression in the spinal cord cervical c-1 has been reported to be associated with AD pathology. Other major AD-related pathways include immune, mitochondrial dysfunction, apoptosis, autophagy, and cholesterol metabolism. Specifically, they are related to the immune system, regulation of Aβ, APP, tau, and other key genes/proteins, autophagy, mitochondrial/endoplasmic reticulum (ER)-mitochondria interactions, mTOR, NFkappaB, estrogens/androgens, helicase/DNA repair, HLA, metal ion metabolism, miRNAs, ROS, p38 MAPK, and VEGF.

We also evaluated the impact of pathway size on our findings, with detailed results listed in Table 3. We observed that two brain regions accounted for a disproportionately large number of the genes analyzed: the cortex (48.34 %) and the hypothalamus (38.44 %). This imbalance was driven by four exceptionally large pathways, two within each of these regions. Specifically, the total number of genes included in the pathways used for ssGSVA is 44,659, with each of these four pathways containing more than 4000 genes. In the cortex, two pathways (GOBP ∼ GO:0008152 ∼ metabolic process and GOBP ∼ GO:0071704 ∼ organic substance metabolic process) originally contain a high number of genes (12,193 and 11,793, respectively), and 3820 and 3664 of them were used in ssGSVA, respectively. Similarly, in the hypothalamus, two pathways (UCSC_TFBS ∼ TBP and UCSC_TFBS ∼ CEBPA) also originally contain a high number of genes (5762 and 4661, respectively), and 1235 and 977 of them were used in ssGSVA, respectively. The rest of the pathways in these two brain regions contain several to several tens of genes (totaling 1058 in the cortex and 1220 in the hypothalamus), which is similar to other brain regions. Additionally, the nucleus accumbens basal ganglia has the highest proportion of AD-related genes (20.03 %).

### 2.5 Quantitatively investigate the interaction relationships among pathways

**Fig. 4:**
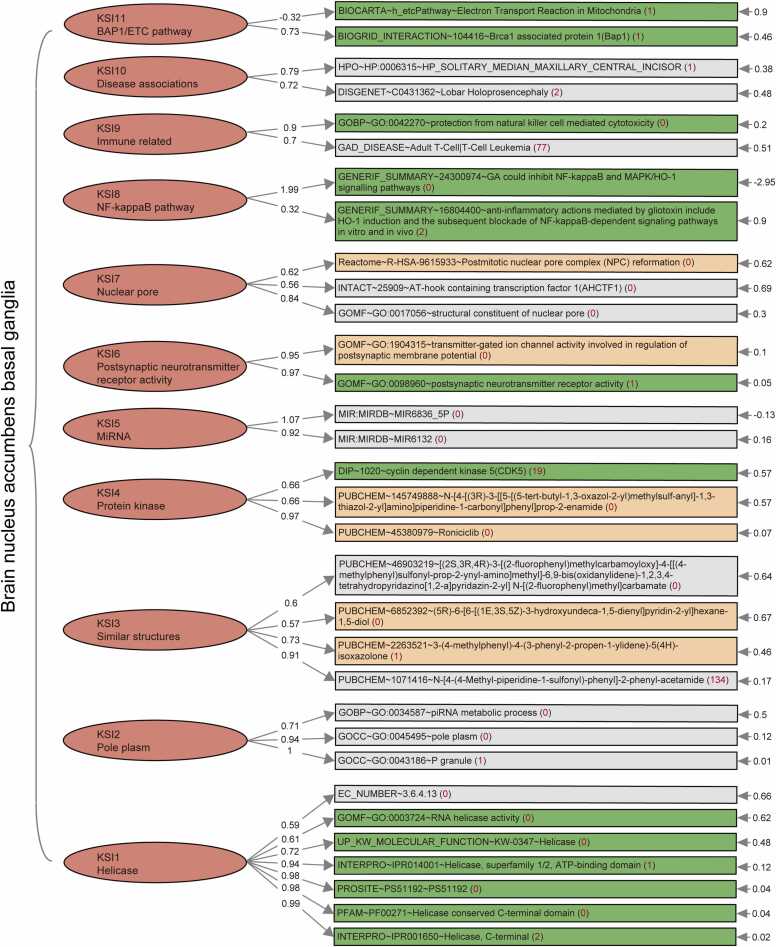
The SEM result of nucleus accumbens basal ganglia. The red numbers in parentheses represent number of pathways with the same ssGSVA score as this pathway in this model. The inter-factor correlations in this brain region can be found in Supplementary Table S13.

Given that pathways do not work independently [36], [37], we utilized SEM analysis to quantitatively assess their interactions and contributions to the phenotype. This process involved factor analysis, model building, and optimization using AD risk-related pathways with unreplicated ssGSVA scores. As shown in Table 4, the Bartlett test for all 14 brain regions was significant after removing variables with inadequate loadings and those with only one variable in a factor. Additionally, the Kaiser-Meyer-Olkin (KMO) values exceeded 0.5 for most brain regions (except for the anterior cingulate cortex BA24, frontal cortex BA9, and pituitary), indicating the appropriateness of factor analysis. After model optimization, the standard error of pathway loading in all brain regions within their respective factors was significant (generally t value > 2), except for the anterior cingulate cortex BA24. The modification indices for pathways in other factors remained relatively low, indicating a generally well-fitting model. Except for the anterior cingulate cortex BA24, the key results of the SEM models for the other 13 brain regions (including pathway attribution factors, loadings, and inter-factor correlations, as well as the corresponding pathways and their associations with AD) can be found in Supplementary Table S5 to S17. Table 4 lists the number and proportion of literature-reported AD-related pathways in each brain region model. Consistent with the proportion of gene counts (Table 3), the model for the nucleus accumbens basal ganglia also contains the largest number of AD-related pathways. Additionally, Fig. 3**F** shows the proportion of direct, indirect, and unrelated pathways within each brain region among all pathways in the SEM results. Fig. 4 provides a detailed overview of the SEM results for the nucleus accumbens basal ganglia, grouping pathways with similar functions into the same factor, regardless of their originating databases. For instance, four pathways, including “anti-inflammatory actions mediated by gliotoxin include HO-1 induction and the subsequent blockade of NF-kappaB-dependent signaling pathways in vitro and in vivo (GENERIF_SUMMARY∼16804400)”, “GA could inhibit NF-kappaB and MAPK/HO-1 signalling pathways (GENERIF_SUMMARY∼24300974)”, “induction of HO-1 by CO-RM2 exerted anti-inflammatory and antioxidant effects which are required in concert to prevent the activation of NF-kappaB leading to induction of various inflammatory genes implicated in the pathogenesis of RA (GENERIF_SUMMARY∼24616552)”, and “Induction of Prx1 by hypoxia regulates heme oxygenase-1 via NF-kappaB in oral cancer (GENERIF_SUMMARY∼25162226)” belong to factor KSI8 within the NFkappaB category. These pathways share the genes HMOX1 (ENSG00000100292) and NFKB1 (ENSG00000109320), which interact and affect AD risk [38]. The inter-factor correlations in this brain region can be found in Supplementary Table S13.

**Table 4:** SEM analysis results of 14 brain regions when using all pathways with the number of genes > 1.

Brain region	KMO	Bartlett	Number of pathways in module	Number of factors	Number of literature-reported AD-related pathways	Percentage
Brain amygdala	0.516	0.000	16	5	1	6.25 %
Brain anterior cingulate cortex BA24	0.483	0.000	NA	NA	NA	NA
Brain caudate basal ganglia	0.588	0.000	44	9	5	11.36 %
Brain cerebellar hemisphere	0.615	0.000	13	3	3	23.08 %
Brain cerebellum	0.617	0.000	541	9	9	1.66 %
Brain cortex	0.632	0.000	551	9	13	2.36 %
Brain frontal cortex BA9	0.493	0.000	12	5	3	25.00 %
Brain hippocampus	0.539	0.000	14	6	5	35.71 %
Brain hypothalamus	0.607	0.000	44	11	8	18.18 %
Brain nucleus accumbens basal ganglia	0.649	0.000	275	11	78	28.36 %
Brain putamen basal ganglia	0.506	0.000	20	8	6	30.00 %
Brain spinal cord cervical c−1	0.516	0.000	13	6	6	46.15 %
Brain substantia nigra	0.506	0.000	11	4	2	18.18 %
Pituitary	0.499	0.000	8	4	3	37.50 %

Number of pathways in module: pathways in module including those with replicated ssGSVA score. Percentage: Number of literature-reported AD-related pathways/Number of pathways in module. More detailed results of each brain region can be found in Supplementary Table S5-S17**.**

## 3 Discussion

In this study, we predicted transcriptomes of 14 brain regions based on genotype data. We then combined these transcriptomes with GWAS summary data to estimate the PRS for AD risk. Subsequently, we characterized the primary inheritance pathways associated with AD. This framework for identifying disease-related pathways offers three main advantages: (1) By starting from genome data, we avoided environmental factors' interference, yielding results that more accurately reflect the disease's intrinsic genetic mechanisms. (2) Our approach relied solely on genotype data, eliminating the need to divide samples into disease or control groups. This enabled us to analyze continuous variables, which provide a more nuanced representation of the real-world disease landscape. (3) Our research covered the entire genome without imposing an arbitrary threshold, preventing the loss of important genomic information.

By translating genetic evidence into gene expression, our approach can circumvent non-genetic confounding factors, a strategy similarly applied by Chen et al. (2021) [39] in their recent study on late-onset AD. The number of genes predicted by GReX is relatively high in the cerebellum/cerebellar hemisphere and pituitary, but their correlation with other brain regions is low. This is consistent with the eQTL results, indicating a higher prevalence of genes exhibiting allele-specific expression in these tissues [21], [40]. The correlation between predicted and measured gene expression levels by RNA-seq was low in the cerebellum region. This is most likely due to the inherent performance limitation of the model. The cross-validated prediction R2 (pred_perf_r2) showed that the prediction model performance of many genes is inherently very limited for all brain tissue in GTEx v8. A study using GTEx v8 data found that for some tissues, the median 5-fold cross-validation R2 for all genes was as low as about 0.04 [41]. The significant positive correlation between the proportion of pathways with high-ratio quantified genes and the percentage of identified AD-related pathways also suggests that more comprehensive eQTL data can enhance the efficiency of our framework.

The trend of pathway numbers enriched by ssGSVA aligned with the predicted gene numbers. The PRS reflected the genetic risk for AD and showed a significant correlation with AD/control grouping, as well as the Braak and Thal stages of the samples. Overall, the higher the Braak and Thal stages, the greater the AD risk. There is a significant difference in pathway GSVA score between the AD/high AD risk group and the contra/low AD risk group. Therefore, we further screened pathways significantly associated with AD risk score. Through correlation analysis, we identified pathways associated with AD risk in each brain region. Of these pathways, 19.7 % have been previously reported to be related to AD, while 39.7 % may potentially be associated with AD. Both PRS and ssGSVA scores are derived from genomic data, allowing us to elucidate AD-related pathways purely from a genetic perspective. Further, the SEM analysis enabled us to assess the interaction and coefficient among pathways. Pathways assigned to one factor tended to share more genes and exhibit similar functions.

**Fig. 5:**
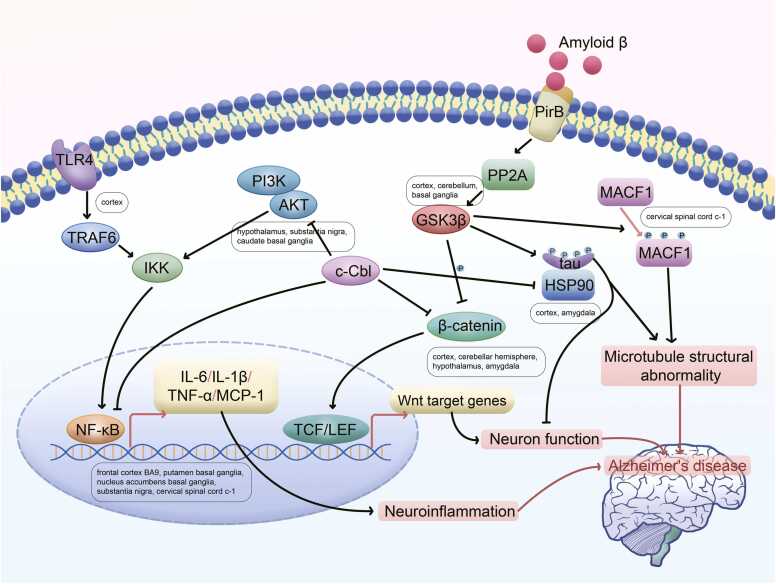
A pathway network summarized by consolidating several AD-related pathways that we have identified.

Research on AD encompasses various aspects such as immune regulation [42], autophagy [43], [44], mitochondrial function [45], and more. The AD-related pathways identified across 14 brain regions span multiple domains, highlighting their significance. Use commonly used or well-annotated pathways or use all genes, the pathways identified as AD risk related primarily address six core biological themes: (1) neuroinflammation and glial dysfunction, as ubiquitous and key drivers; (2) synaptic failure and neurotransmitter system dysregulation, as direct molecular correlates of cognitive impairment; (3) breakdown of cellular proteostasis, involving protein misfolding, ubiquitin-proteasome system (UPS) dysfunction, and impaired autophagy-lysosome pathways; (4) metabolic disturbances and bioenergetics, centered on mitochondrial dysfunction and oxidative stress; (5) genomic instability, manifested by DNA damage accumulation and abnormalities in repair pathways; and (6) dysregulation of key signaling cascades, such as the RAS/MAPK and PI3K/Akt pathways, which integrate and amplify the aforementioned pathological processes. As illustrated in Fig. 5, reviewing these pathways and the literature reports related to AD, we noticed complex crosstalk and regulation between brain regions. Specifically, the accumulation of Aβ, the well-known foundation of AD pathogenesis, serves as a core trigger, activating multiple signaling pathways, ultimately leading to neuroinflammation, neuronal dysfunction, and microtubule structural abnormalities, all contributing to the pathological progression of AD. The transcription factor NF-κB signaling can be activated through the TLR4/TRAF6/IKK or PI3K/AKT signaling pathways, promoting the transcription of cytokines such as IL-1, TNF-α, and chemokines like MCP-1 [46], [47]. This activation stimulates neuroinflammation in cells such as microglia, contributing to the pathogenesis of AD. Amyloid β can transmit signals by binding to its membrane receptor PirB, which then activates GSK3β via PP2A [48]. GSK3β, acting as a multifunctional factor, promotes the phosphorylation of tau and MACF1, leading to their detachment from microtubules, resulting in abnormal microtubule structures and neuronal damage [48], [49]. Additionally, it promotes the degradation of β-catenin by phosphorylation, inactivating the Wnt signaling pathway and impairing neuronal development and function [50], [51]. HSP90 binds to phosphorylated tau and restores its normal structure by dephosphorylation. Abnormal ubiquitination of the protein c-Cbl is closely associated with AD, as it can ubiquitinate and degrade proteins such as AKT, NF-κB, β-catenin, and HSP90 [52], all of which are involved in the occurrence of AD.

Based on our previous research on AD and genomic data [53], [54], we constructed this analysis pipeline and hope that our results can provide some theoretical basis for the study of AD or other complex diseases. However, some inevitable limitations still exist. The number of genes included in a pathway is worthy of consideration when performing enrichment analysis. Given that the predicted number of genes ranges from about 5000 to 10,000, we suggest that the upper limit of the number of genes in a pathway can be set at 300 (∼5 %). Further research is needed to confirm this parameter. Furthermore, ssGSVA only considers genes present in the expression matrix, which can result in pathways containing different genes having identical scores. While randomly selecting a representative for pathways with identical scores during SEM model construction is unlikely to bias the main findings, we cannot completely rule out subtle influences on SEM factor loadings. This handling of identical scores is a limitation of our current method, and future improvements in gene expression prediction may address this issue, further improving SEM accuracy. In addition, manually searching and reading literature takes a lot of time, and artificial intelligence (AI) tools should be used to improve retrieval efficiency and accuracy. On the other hand, we try to avoid using data affected by confounding factors at every step, but we cannot avoid using existing GWAS summary data when making disease risk predictions. Although this data obtained from disease phenotypes may be affected by confounding factors, to our knowledge, this is the least affected option.

In conclusion, given the numerous alterations observed in various pathways in AD, targeting a single process will likely prove insufficient to halt disease progression. More importantly, our findings have pinpointed specific categories that could serve as a reference for investigating pathway-dependent AD risk, offering valuable quantitative insights into the interdependence or compensatory effects among pathways. Further research is warranted to explore the precise roles of pathways and their interrelationships with AD.

## 4 Methods

### 4.1 Whole genomic data

The WGS data from the MayoRNAseq study were utilized in this study. All samples passed data QC as described. Based on the phenotypic information provided, the samples were diagnosed according to the NINCDS-ADRDA criteria and the neuropathologic evaluation [55]. All individuals diagnosed with AD had an empirical diagnosis and were in Braak NFT stage IV or greater. For the control subjects, their Braak NFT stage was III or less, and they exhibited CERAD neuritic and cortical plaque densities of 0 (none) or 1 (sparse). Additionally, the control group did not have any other pathological diagnosis that could potentially confound the AD-related results. We selected samples with a description of white and a diagnosis of AD or control for the following analysis. The genotype profile data of the selected samples were then processed into the format required for gene expression prediction and PRS analysis using plink 1.9 [56]. Referring to Below et al. [39], we performed PCA using plink software and examined the underlying population structure using the Tracy–Widom statistic of the EIGENSOFT software package.

### 4.2 Genome-wide gene expression prediction

The gene expression patterns in different brain regions of individuals were assessed based on GReX approach from the MetaXcan project (https://github.com/hakyimlab/MetaXcan) [22]. To achieve this, we combined the genotype data of MayoRNAseq and eQTL data of GTEx (v8) to establish associations between gene expression and genetic variation in 14 brain regions through linear regression modeling. Subsequently, we used the GReX model with its default parameters to calculate the gene expression in these brain regions. Pairwise correlations between brain regions were calculated using the average gene expression for each region. In addition, the correlation between predicted expression and RNA-seq expression was calculated by the Spearman method using RNA-seq data of the cerebellum region from MayoRNA cohort.

### 4.3 Gene-pathway association collection and ssGSVA

We build a comprehensive, yet non-redundant, knowledge base that integrates established, manually curated biological pathways with broader, interaction-based networks to maximize our discovery potential for complex diseases like AD. We integrated the mapping relationships between pathways and genes from several authoritative databases. Since the pathway data were collected in various formats, we converted them into the list format necessary for conducting ssGSVA. First, we filtered out pathways from various databases that encompassed multiple species, including PathBank (https://pathbank.org/) [32], DAVID (v.2022q2) (https://david.ncifcrf.gov/) [27], NetPath (http://netpath.org/) [29], Reactome (v81) (https://reactome.org/) [28], WikiPathways (v.20220710) (https://classic.wikipathways.org/index.php/) [31], GSEA (MSigDB v7.5.1) (https://www.gsea-msigdb.org/gsea/index.jsp) [26], and PANTHER (http://www.pantherdb.org/) [30]. Only the human pathways were exclusively selected for further analysis. Second, all gene names or IDs were converted to Ensembl gene IDs. Pathway names were organized in the format of type ∼ ID ∼ term (e.g., GOBP ∼ GO:0000002 ∼ mitochondrial genome maintenance). Some pathways were in type ∼ term format if they did not have IDs. Additionally, same pathway in different databases was updated to the latest version. Finally, the curated data were organized into lists of gene sets for ssGSVA. We integrated the pathway information into the Rdata format and stored them at https://github.com/BFGBgroup/Pathway/blob/main/geneSets.Rdata. The R package ‘GSVA’ was used to calculate the score of each pathway in different brain regions for individuals. The number of expressed genes in the pathways was first determined, and pathways containing more than 1 gene or more than 1 and less than 301 genes were further screened.

### 4.4 PRS analysis

The GWAS summary statistics data used in this study were obtained from the research of Jansen *et al.*, including 71,880 AD cases and 383,378 controls [33]. The data were downloaded from a public database (https://ctg.cncr.nl/software/summary_statistics/). For PRS analysis, standard quality control of GWAS summary data and genotype profile data was performed according to the guidelines for PRS analysis [57]. PRScs was then used to calculate the posterior SNP effect size for each chromosome, and the output files from all chromosomes were concatenated [58]. The 1000 Genomes Project phase 3 European subset was employed as the LD reference panel [59]. Finally, the individual-level polygenic score was generated using plink 1.9 [56]. Correlations between PRS and phenotypes (AD/control, Braak NFT stage, Thal) were calculated using the Spearman method.

### 4.5 Correlation analysis between PRS and ssGSVA score

The Shapiro-Wilk test was used to assess the normality of pathway ssGSVA scores. Prior to analysis, outliers were adjusted using the winsor function from the R package psych. The R package ‘HHG’ was employed to calculate the correlation between ssGSVA scores and PRS [34], [35]. To identify pathways significantly associated with the PRS and to correct for multiple comparisons, an adaptive permutation procedure was employed. Specifically, for each pathway, 1000 to 10,000 permutations were performed by randomizing the PRS and ssGSVA scores. The procedure for a given pathway was stopped once the observed *P* value was exceeded by at least 15 permuted *P* values. The threshold for each pathway was determined as follows: First, the empirical *P* value of each pathway was defined as the ratio of occurrences of extreme events (permuted *P* values smaller than the nominal *P* value) to the total number of permutations. Then, for each brain region, the empirical *P* values were corrected to empirical q-values using Storey's approach. The empirical *P* value that corresponded to the empirical q-value closest to 0.05 was selected. Finally, the threshold for each pathway was set as the nth permuted *P* value in ascending order, where n represents the ceiling integer of multiplying the selected empirical *P* value for the brain region by the number of permutations for each pathway.

### 4.6 SEM analysis

To quantitatively investigate the interactions among multiple pathways, we conducted a SEM analysis. Because factor analysis in SEM does not allow for identical variables, we temporarily randomly excluded pathways with identical ssGSVA scores from the analysis. It's important to note that the pathways temporarily excluded from the model input were not discarded from our overall interpretation. First, we used SPSS software (V26.0) to determine the relationship between pathways and factors. Specifically, we obtained the factor loading matrix using principal component analysis (PCA). The number of factors extracted was based on those with eigenvalues greater than 1. We examined the factor loading coefficients in the rotated factor matrix, and variables with inadequate loadings were removed. Specifically, variables were deleted if their loadings on different factors were all below 0.6. This deletion process was repeated until all variables had meaningful associations with the factors. Additionally, factors with only one variable were removed.

Subsequently, the model was constructed and evaluated using LISREL software (V8.7). The model was built based on the Spearman correlation coefficient matrix between pathways and the correspondence between pathways and factors. Modification indices were used to guide the model development. Model improvements were made by considering these indices and the significance of loadings. Specifically, the standard error of the loading of the pathway within their respective factors was considered significant (generally t value >2), and the modification indices for the pathways in other factors remained relatively low. Once the model was finalized, we added the pathways that were temporarily excluded from the model input back to the model results.

### 4.7 Validation with literature evidence

We manually retrieved and summarized AD-related pathways from the literature that were validated by high-throughput data analysis or experiments. To determine the association between identified pathways and AD, we performed an extensive manual literature review. This process involved systematic searches in databases including PubMed and Google Scholar, using keywords such as the pathway name, its critical genes, and Alzheimer's Disease. We defined an association based on published evidence directly linking a pathway to AD or strong evidence linking its key genes to other established AD-related genes or pathways. To ensure the reliability of our findings, all curated associations were independently reviewed and confirmed by multiple team members. These correlations were classified as direct, indirect, or unrelated. "Direct" refers to a direct association of the pathway or factors within the pathway with AD. "Indirect" means the pathway or its factors are correlated with a specific feature that is linked to AD. Other pathways were deemed unrelated to AD.

## CRediT authorship contribution statement

**Jia Wang:** Validation. **Yijie He:** Visualization, Resources, Data curation. **Yaqin Tang:** Visualization, Software, Resources. **Yongheng Wang:** Writing – original draft, Resources, Investigation, Formal analysis, Data curation. **Yingxiong Wang:** Supervision, Conceptualization. **Taihang Liu:** Visualization, Methodology, Investigation, Formal analysis, Data curation. **Zhijie Han:** Writing – review & editing, Validation, Supervision, Methodology, Investigation, Formal analysis, Conceptualization. **Lin Huang:** Visualization, Validation, Conceptualization. **Dongyu Huang:** Visualization. **Pengcheng Tan:** Visualization, Validation, Software. **Zeng Jie:** Visualization, Resources. **Tong Wen:** Visualization. **Lizhen Shao:** Validation.

## Consent statement

The study procedures received approval from the Chongqing Medical University Institutional Review Board.

## Funding

This research is financially supported by the Science and Technology Research Program of National Natural Science Foundation of China (Grant No. 32200446), CQMU Program for Youth Innovation in Future Medicine (Grant No. W0147), and 10.13039/501100007957Chongqing Municipal Education Commission (Grant No. KJQN202100402).

## Declaration of Competing Interest

The authors declare that they have no known competing financial interests or personal relationships that could have appeared to influence the work reported in this paper.

## Data Availability

The data used in this study include MayoRNAseq whole-genome sequencing variant call formats (https://www.synapse.org/#!Synapse:syn11724002), models of gene expression for use in GReX (https://zenodo.org/records/3842289#.YrvrM7FBVYA), and genome-wide summary statistics: (https://ctg.cncr.nl/software/summary_statistics/). We used publicly available software for all analyses, including Plink 1.9 (https://www.cog-genomics.org/plink2/), PRScs (https://github.com/getian107/PRScs), PRS tutorial (https://choishingwan.github.io/PRS-Tutorial), and MetaXcan (https://github.com /hakyimlab/PrediXcan and https://github.com/hakyimlab/MetaXcan/wiki/Individual-level-PrediXcan:-introduction,-tutorials-and-manual). Our framework is stored in Github: https://github.com/BFGBgroup/Pathway/tree/main.

## Footnotes

Appendix A Supplementary data associated with this article can be found in the online version at doi:10.1016/j.csbj.2025.09.013.
